# Prenatal Methylmercury Exposure and Developmental Outcomes: Review of the Evidence and Discussion of Future Directions

**DOI:** 10.1289/ehp.7859

**Published:** 2006-01-18

**Authors:** Anne Spurgeon

**Affiliations:** Institute of Occupational and Environmental Medicine, University of Birmingham, Edgbaston, Birmingham, United Kingdom

**Keywords:** developmental outcomes, methylmercury, neurobehavioral tests, prenatal exposure

## Abstract

I conducted a review of the published literature to assess the strength of the evidence for an association between prenatal exposure to methylmercury (MeHg) and subsequent child development. I identified 12 studies on this subject published since 1980. Of these, 3 were longitudinal studies—2 conducted in the Seychelle Islands, and 1 in the Faroe Islands. Nine were cross-sectional studies conducted in different countries where seafood, a source of MeHg, constituted a major part of the diet. The ages of the children studied ranged from 2 weeks to 12 years. The results of the longitudinal studies were contradictory. Researchers in the Faroe Islands identified an association between MeHg exposure and developmental effects, whereas those in the Seychelle Islands identified no such association. This inconsistency was mirrored in the results of the cross-sectional studies where there were some positive and some negative findings. It was concluded that it was not possible from currently available data to determine whether there is an association between prenatal MeHg exposure and adverse developmental effects in children. In advance of future research, consideration should be given to resolving the uncertainties surrounding exposure assessment and outcome measurement, as both elements varied between studies. It was suggested that questions of exposure assessment would benefit from the application of an expert review process. Outcome assessment would benefit from the development of theoretically based measures of specific aspects of cognitive functioning to replace the relatively crude measures of attainment and IQ currently employed in most studies. This would assist in the development of classic longitudinal studies by allowing repeated assessment over the full age range and providing data that are more readily interpretable and comparable between studies.

Organic compounds of mercury have a variety of industrial uses, and most of the data relating to the neurotoxic effects of mercury exposure have been derived from occupational populations (Chipman et al. 1995). However, in the 1950s a potential risk to the wider community was highlighted when large numbers of people living in the Minamata Bay area of Kyushu Island, Japan, developed symptoms of mercury poisoning. This well-documented incident was traced to a factory using mercuric chloride as a catalyst in the production of vinyl chloride and discharging effluent into the sea (Harada 1995). In aquatic environments, mercury is methylated by the action of common bacteria and methylmercury (MeHg), then passes up the food chain and becomes concentrated in fish and sea mammals. The heavy dependence of the Minamata inhabitants on a fish diet meant that they were subject to high levels of MeHg exposure. Both marine and freshwater fish routinely contain low levels of MeHg as a result of mercury leaching from the earth’s crust. Cases of frank poisoning have not been recorded in fish-eating populations other than those in Minamata, where special circumstances prevailed. However, the Minamata findings raised questions about whether less severe neurologic effects might occur at lower levels of exposure in populations whose diets were rich in seafood.

Given the high level of fetal abnormalities observed in Minamata (Harada 1995), particular concern focused on the potentially hazardous effects of prenatal exposure. This concern increased after an outbreak of mercury poisoning in Iraq in 1971–1972, when large numbers of people consumed bread made from grain treated with a mercury-based fungicide. Subsequent neurologic examination of 80 children born to mothers who had eaten the affected bread over a 2- to 3-month period suggested an increase in neurologic abnormalities (Amin-Zaki et al. 1974). Animal and other data have indicated that the developing fetus is more vulnerable both to exposure to neuro-toxicants such as heavy metals and to the effects of such exposure (Hanson 1997). Any adverse effects are likely to be structurally and functionally different from those seen in the exposed adult. Thus, in addition to the obvious concern for the health of those with diets high in seafood, this issue has wider public health implications in terms of the general advisability of consuming seafood during pregnancy.

The systematic investigation of potential developmental effects in children after prenatal exposure to MeHg began in the 1980s. Since that time two major longitudinal programs of work and a number of smaller cross-sectional studies have been conducted. These investigations assessed levels of prenatal exposure to MeHg in seafood-eating populations and examined subsequent developmental outcomes in children of varying ages. However, despite considerable research effort over a period of 20 years, there remains a lack of consensus on the central issue of whether exposure to MeHg derived from the routine diets of pregnant women presents a risk to their unborn children. This article contributes to the current debate on this subject with a brief description of the currently available data, some possible explanations for the inconclusive results that have so far emerged, and suggestions for potential ways forward in this contentious area of research.

## Current Evidence

A search of relevant databases [Medline (http://omni.ac.uk/medline), Toxfile (http://www.dialog.com), Embase (http://www.embase.com), Cancerlit (http://cancerweb.ncl.ac.uk/cancernet/cancerlit/), Biosis Previews (http://www.biosis.org.uk), SciSearch (http://www.bids.ac.uk), and the Web of Science (http://wok.mimas.ac.uk)], using keywords such as “methylmercury,” “prenatal exposure,” “child development,” and “neurobehavior,” revealed that 12 investigations have been published thus far on this subject, all of which have been conducted since 1980.These investigations have used either neurologic examinations, developmental rating scales, or psychological tests to evaluate postnatal neurologic effects in children prenatally exposed to MeHg. Nine of the studies were cross-sectional (Table 1), in the sense that a sample of children was tested on one occasion and associations between test results and a measure of prenatal exposure to MeHg were examined. (Cordier et al. 2002; Grandjean et al. 1999b; Kjellstrom 1991; Marsh et al. 1995b; McKeown-Eyssen et al. 1983; Murata et al. 1999b; Steuerwald et al. 2000; Stewert et al. 2003; Weihe et al. 2002). Of these cross-sectional studies, however, one consisted of a form of follow-up, in that children were tested at 6–7 years of age and divided into comparison groups on the basis of exposure data collected from their mothers soon after birth (Crump et al. 1998; Kjellstrom 1991). In addition the study of Stewert et al. (2003) comprised a cross-sectional element of a larger longitudinal investigation concerned primarily with the adverse effects of prenatal exposure to polychlorinated biphenyls (PCBs). During the course of this investigation, possible interactions between PCB and MeHg exposure as determinants of adverse health effects were also investigated. Sample sizes in the cross-sectional studies ranged from 43 to 351 and response rates from 64 to 99% (Table 1).

Three studies were longitudinal in design (Table 2) and followed children from birth, at intervals, for several years. Of these, the Faroe Islands study (Budtz-Jorgensen et al. 1999, 2000, 2002; Grandjean et al. 1992, 1995, 1997, 1998, 1999a, 2001a, 2001b, 2002a, 2002b, 2003; Murata et al. 1999a, 2002a, 2002b; Weihe et al. 1996) commenced in 1986 with a cohort of newborn infants who were subsequently tested at ages 12 months, 7 years, and 14 years. Two further longitudinal studies were carried out in the Seychelle Islands. The first commenced in 1987 with a sample of newborn infants who were followed up at age 5 weeks, 66 months, and 108 months (Cernichiari et al. 1995a; Davidson et al. 2000; Marsh et al. 1995a; Myers et al. 1995a, 1995b; Shamlaye et al. 1995). This was considered a pilot study by the authors, although a large amount of data was collected of a good standard and was therefore reported. A subsequent study commenced in the Seychelle Islands in 1989 with a sample of newborns who were followed up at ages 6.5 months, 19 months, 29 months, 66 months, and 108 months (Axtell et al. 1998, 2000; Cernichiari et al. 1995b; Cox et al. 1999; Crump et al. 2000; Davidson et al. 1995a, 1995b, 1998, 1999b, 2001; Myers et al. 1995c, 1995d, 1997, 2000, 2003; Palumbo et al. 2000). Sample sizes in the longitudinal studies were larger than those in the cross-sectional studies (Tables 1 and 2), although there was inevitable attrition over the periods of longitudinal investigation (Table 2).

### Exposure assessment.

With the exception of the longitudinal Faroe Islands study and the study of Steuerwald et al. (2000), all studies used maternal hair as the vehicle for measuring prenatal exposure. In the case of the two longitudinal Seychelle Islands studies and six of the cross-sectional studies (Kjellstrom 1991; Marsh et al. 1995b; McKeown-Eyssen et al. 1983; Steuerwald et al. 2000; Stewert et al. 2003; Weihe et al. 2002), hair samples were taken from the mothers at parturition. However, three of the cross-sectional studies used hair samples taken from the mother and/or the child at the time the child was tested (Cordier et al. 2002; Grandjean et al. 1999b; Murata et al. 1999b). In the study by Grandjean et al. (1999b), for example, exposure was assessed by reference to MeHg concentrations in maternal hair samples and, where this was not available, by reference to concentrations in the child’s hair at the time of testing. Dietary habits and social circumstances had changed little in the community during the previous years. For this reason maternal hair samples were judged likely to be representative of parturition samples. In addition child and maternal hair concentrations were highly correlated. Thus, where it was necessary to use child hair samples, these were also regarded as representing an adequate proxy measure for maternal exposure at parturition The longitudinal Faroe Islands study and the cross-sectional study of Steuerwald et al. (2000) used samples of cord blood as well as maternal hair to assess levels of prenatal exposure. All studies reported details of quality control measures for the analysis of samples. However, the specific form of mercury included in the measurement was not always clear, and a number of different exposure metrics were employed (Tables 1 and 2).

### Outcome measurements.

A variety of outcome measures were used that included neurologic examination, developmental rating scales, neuropsychological tests, and attainment tests. Although this variety was accounted for partly by the differing ages of the children, necessitating different forms of assessment, variation also occurred between studies in terms of the tests used for children of the same age group. All studies used tests or rating scales that were established, published assessment tools. With the exception of the Seychelle Islands studies, information on testing procedures provided in the published reports was rather limited and therefore difficult to evaluate. All studies reported control of some potential effect modifiers such as socioeconomic status, ethnicity, and parental IQ, but the particular factors selected for inclusion varied between studies. All studies used multivariate analysis techniques.

Three of the 12 studies, namely, the longitudinal Faroe Islands study and the studies of Kjellstrom (1991) and Grandjean et al. (1999b), reported a statistically significant relationship between prenatal exposure to MeHg and at least one developmental outcome. In the case of the Faroe Islands study, significant associations were observed for the group as a whole, at 7 years of age, between MeHg in cord blood and some, but not all, of the outcome measures on six neurobehavioral tests. Many of these associations were of borderline statistical significance. However, a dose–response relationship between cord blood MeHg and some test outcomes was demonstrated in a subgroup with the highest MeHg levels. Initial analysis in the Kjellstrom study (1991) did not indicate any significant associations between test outcomes and MeHg. However, exclusion of one outlier whose mother had particularly high MeHg levels resulted in the emergence of associations between MeHg and six test outcomes (Crump et al. 2000; Kjellstrom 1991). In the study by Grandjean et al. (1999b), an association between MeHg exposure was observed for three test outcomes. No discernible pattern was evident in these results in terms of effects on particular tests. Three further studies reported some positive associations between MeHg exposure and developmental outcomes, although all qualify their conclusions and express some reservations about the strength of their findings. In two of these studies, children were under 3 years of age, and assessments were in the form of neurologic examination. Steuerwald et al. (2000) reported that examination of children at 2 weeks of age showed that those with higher prenatal exposures had slightly lower overall neurologic scores, although there was no discernible pattern of suboptimal findings. Similarly, McKeown-Eyssen et al. (1983) carried out neurologic examinations on Cree Indian children between 12 and 30 months of age and found an association between MeHg exposure and the prevalence of abnormal muscle tone reflexes in males only. The authors note the mildness of the abnormality, which they consider to be of doubtful clinical significance. Cordier et al. (2002), in a study of children 9 to 12 years of age, found an association between scores on one test and MeHg exposure, but this did not appear in separate analysis of the highest exposure subgroup. Moreover, results from one test showed a positive association with MeHg exposure. This type of counterintuitive result also occurred in a study of children 7–12 years of age (Weihe et al. 2002) and in the main Seychelle Islands study when children were assessed at 66 months (Davidson et al. 1998). Neither of these studies or the first Seychelle Islands study report associations between other test results and MeHg exposure. Similarly, the results of Marsh et al. (1995b) and Murata et al. (1999b) with children between 6 and 7 years of age indicated no associations between psychological test scores and MeHg exposure. Murata et al. (1999b), however, demonstrated an effect of MeHg exposure on two neurophysiologic measures. Finally, the study of Stewert et al. (2003) of younger children 38 and 54 months of age reported an interactive effect of MeHg and PCB exposure as an incidental finding that emerged in the course of a study to investigate the adverse effects of PCB exposure. A finding of this nature, although interesting, should perhaps be regarded as speculative only and requiring further investigation.

Our relatively brief review of the literature highlights the current uncertainty in this field of enquiry. The two major longitudinal investigations report contradictory findings, and a number of cross-sectional studies have similarly produced inconsistent results. This data set has already been subjected to much examination [National Research Council (NRC) 2000] and further analysis in an attempt to derive appropriate environmental exposure limits for pre-natal MeHg (Office of Environmental Health 1999; Rice et al. 2003). The generally assumed superiority of longitudinal over cross-sectional designs has tended to focus most attention on the studies conducted in the Faroes and the Seychelle Islands, which are of high methodologic quality (Jacobson 2001). However, it should be noted that, given the complex and challenging nature of this type of research, the majority of the cross-sectional studies are also of relatively good quality and would normally be taken into account in a systematic review. The results of these cross-sectional studies in fact mirror the general inconsistency evident in the results of the longitudinal investigations. One is therefore drawn to the conclusion that it is not possible from currently available data to determine whether prenatal exposure to MeHg, at levels routinely experienced by populations whose diets are rich in seafood, results in adverse effects on the nervous system of the developing child.

## Discussion

A primary objective of the review process is the derivation of conclusions from the available data to guide future policy. In this case, however, the development of such a policy would appear to be hindered by the existence of directly contradictory results obtained from research of equal quality. As noted above, the data from the studies contained in this review have already been the subject of extensive evaluation (NRC 2000; Rice et al. 2003) and continue to excite controversy that is so far unresolved (Davidson et al. 1999a; Grandjean and White 1999; Stern and Gochfeld 1999). A secondary objective of the review process, the identification of data gaps in the literature, appears inappropriate in circumstances where so much research has been carried out to date. Although the contribution of existing published research is unquestioned, it may be time to concede that there is little further that can be drawn from these data or, one suspects, from repeated studies of a similar type. Experience from other fields (Spurgeon 2002) suggests that further cross-sectional studies employing similar neurobehavioral outcomes will serve only to increase rather than reduce the uncertainty surrounding this issue. In the remainder of this article, therefore, I discuss some of the possible reasons for the inconsistency in the existing data and indicate some areas where alternative approaches might be required to achieve some progress in this field.

The common objective of the investigations reviewed above was to establish whether there is an association between prenatal exposure to MeHg and developmental effects. Although the various studies had many elements in common, perhaps the most noticeable feature of the studies as a group was the variation in the methods used to assess the two basic elements of the association, namely, the exposure and the effect. It is not surprising that research using different combinations of biological and psychological measures produces inconsistent results. The debate surrounding each of these elements, although undoubtedly complex, merits resolution in advance of any further research.

### Exposure.

In terms of the most appropriate biological marker of prenatal exposure, opinion is divided between maternal hair and cord blood as the biological sample of choice. Studies that have attempted to define the relationship between different biological indices have produced inconsistent and somewhat wide-ranging results, and conversion from one set of values to another appears to involve a number of questionable assumptions (Office of Environmental Health 1999). Other difficulties in the interpretation of the data set arise as a result of the use of different units of measurement and a lack of clarity in some studies about whether the measure is of organic, inorganic, or total mercury concentration. Thus, there is continuing uncertainty about the association between elements of the diet and concentrations in child hair, maternal hair, cord blood, and maternal blood, as well as uncertainty about the strength of any relationship between each of these elements and the relationship between each and the actual exposure of the fetus. Elements of the debate about hair versus blood samples must be linked to a large number of other unanswered questions surrounding prenatal exposure measurement. These relate particularly to the relative importance of exposure at different periods of gestation, the relationship between these and average exposures, and the importance of peak exposures. The development of the central nervous system is time related and unidirectional. The inhibition of one stage of development tends to cause alterations to subsequent processes, with limited capacity for compensation for cell loss (Annau and Eccles 1986; Trask and Kosofsky 2000). Both the dose and timing of any environmental insult are important in terms of the specific nature of any adverse effects. How far do our current methods of prenatal exposure assessment reflect the need to take this into account?

The present enthusiasm for evidence-based policy and practice appears to offer an ideal opportunity to address these types of questions, either through the medium of an expert workshop or that of a written systematic review. The important issues in either process include *a*) definition of the important questions to be addressed to achieve valid and reliable assessment of prenatal exposure, *b*) identification of available data that could be used to answer these questions, and *c*) identification of new research required to fill any identified data gaps. In advance of some consensus on these issues, further research is likely to provoke more controversy rather than lead to any resolution of the current uncertainty.

### Outcomes.

The outcomes used in these studies were predominantly psychological tests. Use of such tests in environmental and occupational health research, which began in the early 1980s, has always been controversial, and the apparent inconsistencies in the data produced has provoked much debate in both environmental and occupational health research (Koller et al. 2004; Levy et al. 2004). Results relating to prenatal MeHg exposure represent a particular example of a wider problem and highlight a number of questions related to the more general field of neurobehavioral toxicology.

Specifically, two main areas are of concern. The first, and perhaps the more straightforward, relates to the control of variables that either represent potential confounders or may act as modifiers of the effects under investigation (Spurgeon and Gamberale 1997). They are perhaps best considered under the broad headings of situational variables (physical testing conditions and test procedures), tester variables (reliability of the examiners), and subject variables (individual characteristics such as age, gender, and socioeconomic group). In all epidemiologic research involving psychological testing, the list of these variables is potentially very long, and researchers appear divided about which to include. In research on MeHg, the majority of studies consider important subject characteristics such as age, ethnic and socioeconomic group, and aspects of parental lifestyle. However, for a number of other variables (e.g., aspects of the caregiving environment), inclusion is patchy. For many of these variables, useful literature is available on their effects on children’s abilities or on test performance, and it may be possible to reach an evidence-based consensus on their inclusion or exclusion. For other, mainly procedural factors, data appear relatively scarce. A systematic review that encompasses other areas of psychology, for example, that pertaining to human–computer interaction, might reveal relevant information. For example, how much does the size of the screen affect performance on a computer-administered test? How much does the physical location of testing (home, laboratory, hospital) affect test performance? Existing data on the effects of time of day (Smith 1992), for example, indicate that in epidemiologic studies this factor should always be controlled. Intuitively it would seem appropriate that the physical testing situation and procedures should be standardized for all subjects as far as is practically possible, regardless of whether firm evidence exists about the influence of heating, lighting, noise control, or the arrangement of furniture. Less well-researched aspects of the test situation can be explored usefully within the researchers’ data. Is there, for example, a significant difference between test scores obtained at the beginning and at the end of the week or at different times of the year?

The effects of the tester, particularly where tests are not computer-administered, may be important, not only because of different interactions with different subjects but also because of the examiner’s variable moods, motivation, levels of fatigue, and tendency to introduce systematic errors into the testing procedure. It cannot be assumed that confining testing to one examiner or using examiners who have undergone a single period of training removes tester variation. In terms of reliability, it may be advantageous to employ more than one tester in some circumstances. Measures such as the videotaping of testing procedures, double scoring, and examination of the test data for trends related to some of these factors have all been used to account for or eliminate this potential source of variation (Harvey et al. 1988). Similarly some estimation, albeit a subjective rating, of the child’s level of co-operation with the testing procedure is important to include. Potentially this is a major source of variation in test performance rarely alluded to in published reports. Ideally, tests should also include parallel forms or practice trials to ensure that maximal performance level is recorded for each subject.

All except one of the studies in the field of research under discussion here present detailed accounts of quality control procedures in relation to MeHg assessment. It is relatively rare to find equally detailed discussion of procedures for outcome assessment. This is a situation that occurs frequently in neurobehavioral investigations. Lack of reference to quality control does not necessarily imply that control was limited but may suggest something about the attitude of researchers toward its importance. The implications for further research are 2-fold. First, systematic work is needed on the effects of factors considered likely to affect test performance, including both a review of the available data and, if necessary, further investigative work. Second, consensus must be reached on good practice such as that available in some other areas of toxicology, notably animal experimentation. Although this consensus may exist at an informal level in the field, the considerable methodologic variations between different neurobehavioral studies suggest that many aspects are currently opinion based rather than evidence based.

A second and fundamental issue in terms of outcome measures relates to the types of tests used and, by implication, the interpretation of the results they provide and the comparability of these between studies. The tests employed in the studies described above are mainly tests of intellectual functioning. However, those used in different studies, and sometimes within the same study, derive from a number of separate traditions of intellectual assessment, each of which was developed for a different purpose and different client group. Although each has some advantages, none were developed specifically for neurotoxicity research and none is entirely appropriate for this type of application.

Attainment tests are attractive in the sense that they offer the opportunity to benchmark the performance of children in basic skills such as literacy and numeracy against that of their peers. However, such tests tend to reflect the use of abilities rather than the underlying abilities themselves. Given the range of social and educational factors interacting with the ability to produce attainment, this effectively introduces additional variables into the equation (Gadzella et al. 1989).

In contrast, the neuropsychological approach characterized, for example, by tests such as the Trailmaking test used in the Seychelle Islands study or the Bender Gestalt test used in the Faroes Islands study was developed to provide detailed evaluation of patients with suspected damage to the brain. Such damage might have resulted from head injury or other insult or from a degenerative disease of the nervous system. In these circumstances the purpose of assessment is to provide detailed information about the nature of the problem in functional terms and thus provide a basis for rehabilitation and progress monitoring. Assessment in a clinical setting tends to be a flexible process that draws as much on the qualitative aspects of the interaction between psychologist and client as it does on the numerical test scores. The clinician is interested in the patient as an individual and reaches a professional judgment on the basis of a number of sources of information. There is a risk that tests of this nature lose much of their value when applied in a routine fashion to large groups of people. Many who work in the field of clinical neuropsychology appear to be deeply uneasy about the transfer of these techniques to an epidemiologic setting (British Psychological Society 2001). Particular concern arises when tests designed for administration by a psychologist are adapted for computer presentation. Researchers with a neuropsychological background tend to adopt a clinical approach by administering a very large battery of tests to cover all aspects of functioning (Davidson et al. 1995a; Grandjean et al. 1997; Kjellstrom 1991). In an epidemiologic setting this can be inappropriate, resulting in multiple comparisons and the possibility of chance findings. Moreover, it often leads to confusion from a psychological point of view, where the results appear as a collection of apparently unconnected findings with no discernible meaningful pattern. Where studies use the same tests, it is common for significant associations to appear in both studies but in relation to different outcomes (Grandjean et al. 1997, 1999b). Finally, there are questions about the ability of tests designed for more severely affected patient groups to detect relatively subtle effects in community samples (Spurgeon 1996; Stollery 1985, 1990).

Tests derived from a psychometric tradition are concerned with the assessment of intelligence quotients (IQ) in the general population and were originally developed to describe normal distributions of cognitive functioning. The Wechsler Scale (Wechsler 1991) represents the most widely used test battery in this respect. Developmental scales for very young children fit within this tradition, replacing formal testing where this is impractical, although it should be noted that maternal reports of developmental milestones are subject to numerous sources of error such as inaccurate recall, differing definitions of certain behaviors, and presentational bias (Axelson and Rylander 1984).

The measurement of IQ is a reassuringly familiar concept supported by a wealth of normative data and experience built up over many years. Unfortunately, IQ tests were originally developed within a theoretical framework of cognitive functioning that prevailed more than a half-century ago. Such tests reflected a contemporary need to categorize individuals on a quantitative scale to predict future performance, an approach now considered somewhat crude and simplistic. Although such tests maintain their predictive validity in some settings (Neisser et al. 1996), they are relatively blunt instruments that combine a number of different abilities within each test (Lezak 1988). This aspect limits the information that can be derived from the assessment and makes interpretation difficult when conflicting results emerge from different studies. When placed in the context of more recent theoretical developments in cognitive psychology, established IQ tests do not provide results that can be easily linked to current models of cognitive processes.

A primary objective of neurobehavioral research is the detection of subtle effects on cognitive functioning in community samples after neurotoxicant exposure. For epidemiologic purposes, tests should be quick and easy to administer. The results should be interpretable at group level and comparable between different studies. Given these criteria, none of the tests currently in use appear to be entirely fit for this purpose. Speed and ease of administration do not represent major challenges in an age of advanced information technology. However, improvements in interpretability and comparability are more complex issues likely to require a radical change of approach. In recent years a number of authors have pointed to the overemphasis on empiricism in this field and the lack of a strong theoretical underpinning for the assessment tools employed (Stephens and Barker 1998; Stollery 1990, 1996; Williamson 1990). The development of tests grounded in well-established cognitive theory would allow results to be discussed in terms of the specific aspects of cognitive processing under investigation rather than simply by reference to broad and largely uninformative categories of effect such as “memory” or “attention.” Modern approaches to the study of memory processes, for example, have long distinguished between several elements that contribute to the final outcome (initial registration of information, encoding, transfer to long-term store, loss of information by decay or interference, and use of cues for retrieval) (Baddeley 1987). Each may be differentially susceptible to neurotoxic insult, but effects on one specific process cannot be uncovered by most current tests that provide a simple global outcome score. Moreover, overall scores may mask specific effects where subjects employ compensatory strategies among different processes to achieve maximum performance. The development of tests, for both children and adults, based on techniques currently available to separate and measure these specific processes would provide much more useful information about the nature and size of any observed effect. This type of approach would ultimately pave the way for much greater comparability between the results of different investigations and for the development of comparable assessment techniques for children at different ages during longitudinal investigations. Despite much international effort during the last 25 years, agreement on a universally approved set of tests has proved elusive (World Health Oganization 1989). At the same time, the pursuit of the goal to achieve comparability over time and between studies has tended to inhibit the development of new techniques. It seems unlikely that consensus on appropriate assessment tools will be achieved in advance of a consensus on the theoretical basis for those tools. Fortunately, much of the information required for these new developments is readily available in the existing cognitive, experimental, and developmental psychology literature.

## Conclusion

Reviews of the data relating to the developmental effects of prenatal MeHg exposure have highlighted the inconsistency of the currently available evidence. The size and nature of the risk to children that is associated with seafood consumption by their mothers remains uncertain and a source of considerable controversy. It has been argued here that the present uncertainty derives from the variation between studies in the methods used to measure both the exposure and the effect. Each element would merit further consideration in advance of any future research in this field. Although consensus is required on the appropriate biological marker of exposure, there is also a particular need to address issues of both procedure and content in psychological assessment. Discussion of these issues, particularly those relating to psychological tests, may have implications that go well beyond the immediate needs of this field of inquiry. Investigation of the effects of MeHg provides one particular example of the difficulties in data interpretation that occur repeatedly in neurobehavioral studies and threaten to undermine confidence in this methodology. The increasing international anxiety about potential adverse effects of low-level neurotoxicant exposure in the environment underlines the importance of addressing these concerns, as psychological methods currently represent one of the main tools of research in this field.

## Figures and Tables

**Table 1:** Cross-sectional studies.

Reference/country	Response rate (%)	*n*	Age at testing	Exposure measure	Exposure level	Test type
Steuerwald et al. 2000 Faroe Islands	64	182	2 weeks	CB, MH	20.4 μg/L 4.08 μg/g	NE
McKeown-Eyssen et al. 1983 Canada	95	234	12–30 months	MH	6 μg/g	NE, DS
Stewert et al. 2003 Canada	72	212	38 months 54 months	MH	0.5 ng/mg	PT
Marsh et al. 1995a, 1995b Peru	NS	131	NS	MH	7.05 ppm	NE, DM
Kjellstrom 1991, Crump et al. 1998 New Zealand	NS	237	6–7 years	MH	> 6 mg/kg 3–6 mg/kg < 3 mg/kg	PT, AT
Murata et al. 1999a, 1999b Madeira	99	149	6–7 years	MH	9.64 μg/g	PT, NT
Grandjean et al. 1999a, 1999b Brazil	84	351	7–12 years	MH	11.6 μg/g	PT
Weihe et al. 2002 Greenland	NS	43	7–12 years	MH	15.5 μg/g	PT, NT
Cordier et al. 2002 French Guiana	88 85 80	97 69 82	9 months–12 years	MH	12.7 μg/g 6.7 μg/g 2.8 μg/g	NE, PT

Abbreviations: AT, attainment tests; CB, cord blood; DM, developmental milestones; DS, developmental scales; MH, maternal hair; NE, neurologic examination; NS, not stated; NT, neurophysiologic tests; PT, psychological tests.

**Table 2:** Longitudinal studies.

Age at testing	Response rate (%)	*n*	Exposure measure	Exposure level	Test type
Faroe Islands					
12 months	57	583	CB, MH	121 nmol/L, 4.5 μ/g	DM
7 years	90	917	CB	114 nmol/L	NE, PT, NT
14 years	86	882	CB	NS	PT, NT
Seychelle Islands (pilot)					
5–109 weeks	98	789	MH	6.6 ppm	DS, NE
66 months	NS	217	MH	7.1 ppm	PT
108 months	NS	87	MH	< 3–> 9 ppm	PT
Seychelle Islands (main)					
6.5 months	95	740	MH	5.9 ppm	VR, NE, DS
19 months	95	738	MH	5.9 ppm	DM, DS
29 months	94	736	MH	5.9 ppm	DS
66 months	91	711	MH	6.8 ppm	PT
108 months	83	643	MH	6.9 ppm	PT, TR

Abbreviations: NS, not significant; TR, teacher rating; VR, visual recognition.
